# Current Models to Study the *Sporothrix*-Host Interaction

**DOI:** 10.3389/ffunb.2022.833111

**Published:** 2022-02-25

**Authors:** Ana P. Vargas-Macías, Manuela Gómez-Gaviria, Laura C. García-Carnero, Héctor M. Mora-Montes

**Affiliations:** División de Ciencias Naturales y Exactas, Departamento de Biología, Universidad de Guanajuato, Guanajuato, Mexico

**Keywords:** pathogen-host interaction, virulence factors, animal model, antifungal drugs, immune response, invertebrate model, *ex vivo* model

## Abstract

Sporotrichosis is a worldwide distributed subcutaneous mycosis that affects mammals, including human beings. The infection is caused by members of the *Sporothrix* pathogenic clade, which includes *Sporothrix schenckii, Sporothrix brasiliensis*, and *Sporothrix globosa*. The fungus can be acquired through traumatic inoculation of conidia growing in vegetal debris or by zoonotic transmission from sick animals. Although is not considered a life-threatening disease, it is an emergent health problem that affects mostly immunocompromised patients. The sporotrichosis causative agents differ in their virulence, host range, and sensitivity to antifungal drugs; therefore, it is relevant to understand the molecular bases of their pathogenesis, interaction with immune effectors, and mechanisms to acquired resistance to antifungal compounds. Murine models are considered the gold standard to address these questions; however, some alternative hosts offer numerous advantages over mammalian models, such as invertebrates like *Galleria mellonella* and *Tenebrio molitor*, or *ex vivo* models, which are useful tools to approach questions beyond virulence, without the ethical or budgetary features associated with the use of animal models. In this review, we analyze the different models currently used to study the host-*Sporothrix* interaction.

## Introduction

Sporotrichosis is a benign subcutaneous mycosis that shows different clinical manifestations, with lesions usually restricted to the skin, subcutaneous tissue, or adjacent lymphatic vessels. It can affect both humans and other mammals. However, in immunocompromised patients, the disease can be a life-threatening systemic or disseminated infection (Barros et al., 2011; López-Romero et al., 2011). The etiological agents are members of the *Sporothrix* clinical clade, which includes *Sporothrix schenckii, Sporothrix brasiliensis*, and *Sporothrix globosa* (de Beer et al., 2016), the most frequently isolated species from both clinical and veterinary cases (Lopes-Bezerra et al., 2018a). These species have different distribution patterns, being *S. brasiliensis* mainly restricted to Brasil and Argentina, and *S. schenckii* and *S. globosa* with a worldwide distribution, but found preferentially in America and Asia, respectively (Chakrabarti et al., 2015; Mora-Montes et al., 2015; Lopes-Bezerra et al., 2018a; Etchecopaz et al., 2020). Sporotrichosis is acquired through traumatic inoculation with vegetal material contaminated with the mycelial morphology, thus being considered as a sapronosis, or through zoonotic transmission by domestic animals, most frequently by cats that inoculate the yeast morphology directly into the host (Mora-Montes et al., 2015; Lopes-Bezerra et al., 2018a). Feline sporotrichosis is mainly associated with *S. brasiliensis* (Rodrigues et al., 2016).

**Figure 1:**
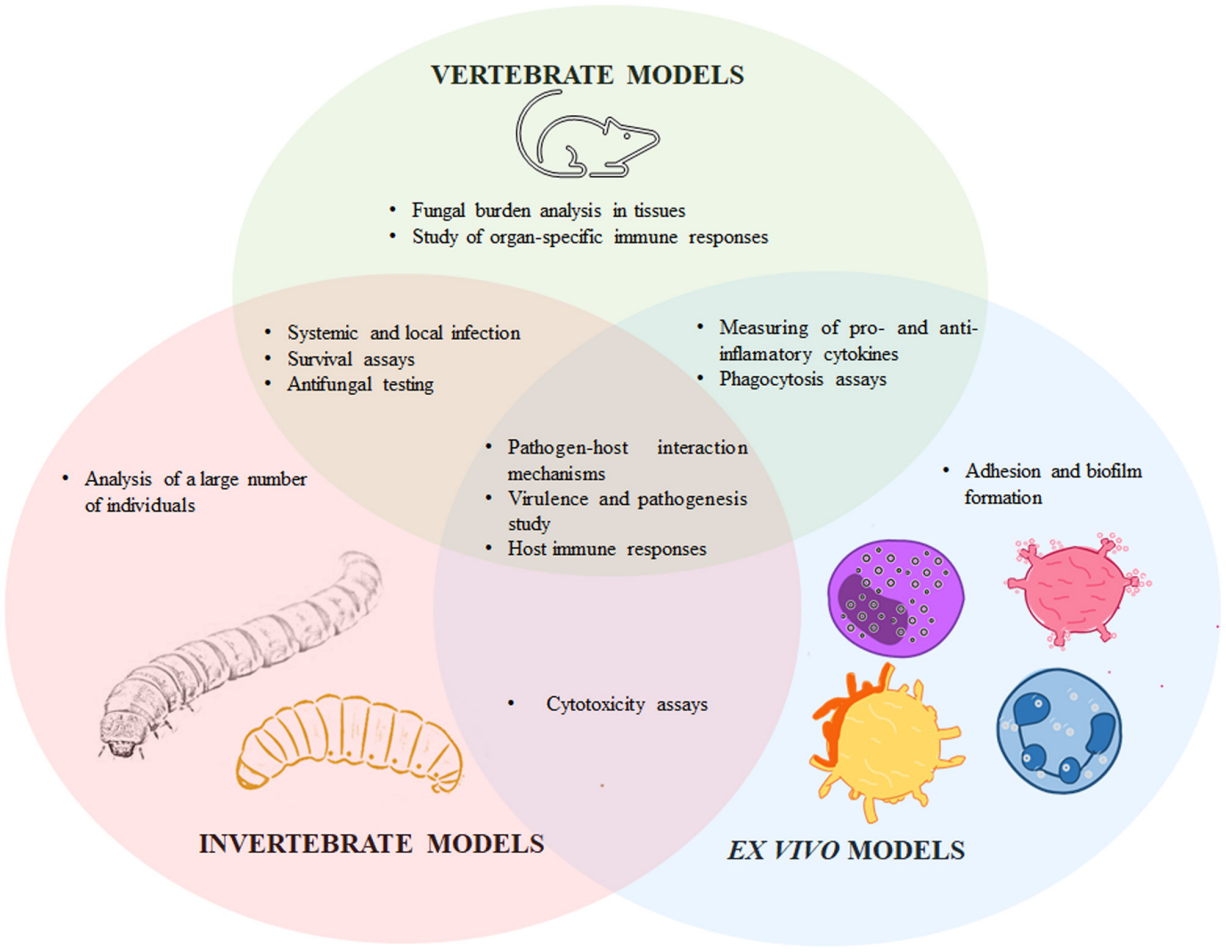
Particularities and similarities of vertebrates, invertebrate, and *ex vivo* models to experimentally study sporotrichosis. The *Sporothrix*-host interaction has been assessed in vertebrate models such as mice, rats, guinea pigs, and cats; invertebrates, including *Galleria mellonella, Tenebrio molitor*, and *Acanthamoeba castellanii*; and *ex vivo* models such as endothelial cells, macrophages, human peripheral blood mononuclear cells, mast cells, dendritic cells, cell lines, and feline claw fragments. These models have particularities that differentiate them from each other, but they also share applicabilities in the study of host-*Sporothrix* spp. interplay.

The study of fungal virulence is critical to understanding the outcome of the host-pathogen interactions, the different disease presentations, the molecular bases of tissue-damaging, and those that allow the hosts' cells to defend themselves, and the ability of the fungal cells to divert or respond against the host's immunity. Therefore, virulence is an essential aspect to take into account during the development of new alternatives for fungal infections treatment and prophylaxis. Traditionally, virulence is analyzed *in vivo*, using laboratory animals that mimic the fungal infective cycle and the host immune response; and mammals, in particular mice, are currently the gold standard to experimentally evaluate sporotrichosis and *Sporothrix* spp. virulence. However, some other models have been proposed, such as *ex vivo* experimental models that use endothelial and immune cells, and non-conventional invertebrate hosts, such as *Galleria mellonella, Tenebrio molitor*, and *Acanthamoeba castellanii*. In this review, some of the main models to study *Sporothrix* spp. virulence will be discussed, underlining the applications of each model (see Figure 1).

## Mammalian Models to Study Sporotrichosis

The study of fungal pathogenesis is traditionally linked to *in vivo* models, to examine virulence factors, the host's antifungal immunity, and the sensitivity and efficacy of antifungal compounds (Hohl, 2014). For this purpose, mammalians are the most used models, and among them, rodents are considered the gold standard for studying pathogenesis and efficacy of antifungal drugs (Chamilos et al., 2007; Binder et al., 2016). Mice are often used to assess the *Sporothrix* spp. virulence using different animal strains, such as BALB/c, C57BL/6, and OF-1; and experimental infection techniques, like intraperitoneal, intravenous, and subcutaneous fungal delivery (Arrillaga-Moncrieff et al., 2009; Carlos et al., 2009; Della Terra et al., 2017). The main advantages of mice models are that their reproductive cycle is relatively short and litters contain several members, allowing the use of various animals per experimental group; the animal manipulation and dissection are well-known and easy to replicate; and genetically modified strains are currently available, allowing the assessment of particular genes in the host-fungus interaction (Clemons and Stevens, 2005). Rats also represent a viable option to study fungal pathogenesis, with advantages similar to those found in mice, but with the main disadvantage that the animal housing is relatively more expensive when compared to mice (Chamilos et al., 2007). In the last century, the use of other mammals, such as guinea pigs and cats was common in clinical investigations, but nowadays the standard choice usually is a murine model (Orofino-Costa et al., 2017). Moreover, the use of animal models was important in demonstrating the different virulence attributes of organisms that seemed to be morphologically similar or identical, that was the case of all the environmental isolates recovered from the biggest sporotrichosis outbreak occurring in New York in 1988. All of the isolates produced *Sporothrix* spp. anamorphs, characterized by conidia arranged in a sympodial form on the inflated apices of lateral conidiophores, but only those that produced melanized conidia and grew up at 37°C produced fatal infections in mice, supporting the mycological identification of *S. schenckii* in a time where DNA typing studies were scarce (Dixon et al., 1991, 1992).

The medically relevant *Sporothrix* species can generate conidia, hyphae, and yeast-like cells (López-Romero et al., 2011; Lopes-Bezerra et al., 2018a), and the fungal morphology used for host inoculation, along with the infection route, and the animal genetic background, should be taken into account during data interpretation. As an example, it was reported that phagocytic receptors on macrophages distinguish between yeast and conidia morphotypes, with different outcomes in the response of these immune cells (Guzman-Beltran et al., 2012). Conidia are often used to analyze the *in vivo* dimorphism to yeast-like cells, but since the latter morphology is considered the pathogenic and invasive morphology, some researcher groups prefer the inoculation of this fungal morphology. This is an ongoing debate in this area that remains to be solved. In addition, instead of cells, soluble antigenic preparations from *S. schenckii* can be isolated and used to inoculate mice (Carlos et al., 1992; Huang et al., 2021). External factors such as culture media, cell longevity, and maintenance conditions should also be considered during the analysis of the host-fungus interaction since they have been reported to influence cell wall composition, fungal morphogenesis, and virulence (Lopes-Bezerra et al., 2018b; Lozoya-Pérez et al., 2020).

The phenotypic and genomic analyses have demonstrated that *Sporothrix* spp. has different virulence degrees, which are associated with particular clinical forms (Arrillaga-Moncrieff et al., 2009). In the mouse model of systemic sporotrichosis, *S. brasiliensis* is the most virulent species of the pathogenic clade, and is associated with several clinical manifestations and high tissue burdens in animal models; while *S. schenckii* generally shows moderate virulence, causing mostly chronic subcutaneous infections (Arrillaga-Moncrieff et al., 2009; Lopes-Bezerra et al., 2018a). *S. globosa* is described as a low virulence species and is mainly associated with lymphocutaneous infections (Cruz Choappa et al., 2016).

The tissue from which a *Sporothrix* strain is isolated may also be involved in fungal virulence. It has been described that *S. schenckii* strains isolated from lymphocutaneous lesions showed more multiplication in the mouse internal organs than strains isolated from fixed cutaneous lesions (Kwon-Chung, 1979). Accordingly, it has also been reported that isolates from cutaneous lesions only produced footpad swelling in mice (Tachibana et al., 1998), and *S. schenckii* strains isolated from human lymphocutaneous sporotrichosis caused a disseminated infection in the murine model (de Capriles et al., 1993). *Sporothrix* isolates intrinsic factors such as pigmentation, capacity to grow at 37°C, and ergosterol peroxide production, influence the virulence in mice (Arrillaga-Moncrieff et al., 2009; Carlos et al., 2009). Melanin protects against phagocytosis and oxidative killing, and it has recently been reported that this pigment can also inhibit the major histocompatibility complex expression on the macrophage surface *in vivo* (Almeida-Paes et al., 2012; Song et al., 2021). Adhesion to fibronectin is another important *Sporothrix* virulence factor. Strains with more adherence to the extracellular matrix components are the most virulent *in vivo* (Teixeira et al., 2009). Surface components of the *S. schenckii* wall have been characterized, and their relevance in pathogenesis has been described. Peptidorhamnomannan is an *S. schenckii* cell wall glycoconjugate composed of rhamnose, mannose, protein, and polysaccharides containing galactose with antigenic capacity, making it important for pathogenesis (Lopes-Bezerra, 2011). The *O*-linked oligosaccharides are also cell wall components that are highly immunogenic and possibly involved during host colonization (Lopes Alves et al., 1994).

In this context, it has been reported that even when using the same strain, the virulence degree can vary according to the route of infection, demonstrating that the intravenous route favors systemic infections and the subcutaneous route mimics the natural infection produced by cat bites or scratches (Brito et al., 2007). In addition, external factors could influence virulence as well, such as the growing conditions of fungal biomass; *S. schenckii* yeast-like cells cultivated in a rich medium such as brain-heart infusion were more virulent on mice than cells grown in poorer culture medium (Teixeira et al., 2010; see Table 1).

**Table 1:** Application of mammalian, invertebrate, and *ex vivo* models in the study of the *Sporothrix*-host interaction.

**Study field**	**Model**			**References**
	**Mammalians**	**Invertebrates**	***Ex vivo***	
Virulence factors	✓	✓	X	Tachibana et al., 1998; Fernandes et al., 1999; Nascimento and Almeida, 2005; Kong et al., 2006; Brito et al., 2007; Arrillaga-Moncrieff et al., 2009; Ruiz-Baca et al., 2009; Teixeira et al., 2009, 2010; Almeida-Paes et al., 2012, 2015; Castro et al., 2013; Clavijo-Giraldo et al., 2016; Martinez-Alvarez et al., 2019; García-Carnero et al., 2021; Song et al., 2021
Host innate response	✓	✓	✓	Shiraishi et al., 1992; Sassá et al., 2009; Guzman-Beltran et al., 2012; Guzman Beltrán et al., 2021; Romo-Lozano et al., 2012, 2014; Sandig and Bulfone-Paus, 2012; Negrini Tde et al., 2013; de Almeida et al., 2017, 2018; Martinez-Alvarez et al., 2017, 2019; Lozoya-Perez et al., 2018, 2019; Lozoya-Pérez et al., 2020; Lozoya-Perez et al., 2021; Rossato et al., 2019; Huang et al., 2021; Tamez-Castrellón et al., 2021
Host adaptative response	✓	X	X	Shiraishi et al., 1992; Tachibana et al., 1998, 1999; Nascimento and Almeida, 2005; Romo-Lozano et al., 2014; Batista-Duharte et al., 2018b, 2020
Antifungal drugs testing	✓	✓	✓	Tsubura and Schwarz, 1961; Kan and Bennett, 1988; Meinerz et al., 2008; Fernández-Silva et al., 2012a; Mario et al., 2015; Borba-Santos et al., 2020, 2021; Passos et al., 2020
Systemic infection	✓	✓	X	Nascimento and Almeida, 2005; Meinerz et al., 2008; Teixeira et al., 2009; Clavijo-Giraldo et al., 2016; Jellmayer et al., 2017; Lozoya-Perez et al., 2018, 2019; Lozoya-Pérez et al., 2020; Lozoya-Perez et al., 2021; Manente et al., 2018; Rossato et al., 2019; García-Carnero et al., 2021; Tamez-Castrellón et al., 2021
Subcutaneous infection	✓	X	X	Tachibana et al., 1999; Kong et al., 2006; Brito et al., 2007; Arrillaga-Moncrieff et al., 2009; Castro et al., 2013; Fernandes et al., 2013; Della Terra et al., 2017; de Almeida et al., 2018

### Systemic and Peritoneal Models of Sporotrichosis in Mice

In general terms, in an experimental model of systemic sporotrichosis, mice can be inoculated intraperitoneally or intravenously either in the tail vein or in the retro-orbital plexus (Tachibana et al., 1998; Fernandes et al., 1999). For a disseminated fungal infection, the criterion for virulence is usually the measurement of median survival times; however, after infection, inactivity signs and weight measurements can be daily recorded, and blood samples can be collected to evaluate the levels of circulating immune mediators such as cytokines and antibodies. The fungal load can also be determined by quantifying the colony-forming units in the kidneys, lungs, spleen, brain, testicles, and popliteal lymph nodes; and histopathological studies in different organs can be assessed as well (Arrillaga-Moncrieff et al., 2009; Fernández-Silva et al., 2012b; Manente et al., 2018).

Most of our knowledge about the host immune response against *Sporothrix* spp. has been determined using the model of systemic infection in mice (Martinez-Alvarez et al., 2014). The importance of the host immune responses for the sporotrichosis control is evidenced by the higher fungal colonization and dissemination in the immunosuppressed mouse model and immunocompromised patients (Batista-Duharte et al., 2018a; Manente et al., 2018). Thus, the comparative study of systemic sporotrichosis using both immunocompetent and immunosuppressed animals may contribute to understanding the disease immunopathogenesis, searching for new prophylactic or therapeutic options, and evaluating xenobiotics effects on the infection etiological agent. To understand the immunological mechanisms involved in sporotrichosis prevention and control, mice were inoculated in the footpad with *S. schenckii* cell wall soluble antigens. Eight weeks after inoculation, it was observed an increase of mononuclear cells, as well as paw thickening, which could be due to a delayed hypersensitivity reaction (Carlos et al., 1992). Given that high titers of antigens were used in this experiment, immunological tolerance might be induced in the host, being reflected in a depression of cellular immunity (Carlos et al., 1992). Moreover, it has been already reported the importance of cell-mediated immunity in the protection against *S. schenckii* infection (Shiraishi et al., 1992). Experiments using nude mice also support the importance of CD4+ T cell-mediated immunity against *S. schenckii*. Congenitally athymic mice were found to be more susceptible to intravenous infection with *S. schenckii* than normal mice, based on lethality and measurement of viable yeast-like cells in the liver after 7 days of infection (Dickerson et al., 1983; Kajiwara et al., 2004). In line with these observations, thymus transplantation from normal to nude mice conferred a significant degree of protection, suggesting that thymus-derived cells play an important role in the resistance against sporotrichosis (Dickerson et al., 1983). Nude mice intratestically infected had a higher fungal load in testis than wild-type animals, supporting the idea that T cells play a role in the inhibition of fungal growth in organs (Shiraishi et al., 1992).

As mentioned, one advantage of murine models is the availability of knockout mice in several immunity-related genes, and thus, it is possible to assess their contribution during the establishment of the infection caused by *Sporothrix* spp. When the responses of wild-type and tlr4 ^−^/^−^ knockout mice against intraperitoneally inoculated *S. schenckii* cells were compared, it was found that nitric oxide, tumor necrosis factor-alpha (TNF-α), and interleukin (IL)-10 levels were significantly reduced in the knockout mice, suggesting a possible role of this receptor in the recognition of *S. schenckii* yeast-like cells (Sassá et al., 2009). These observations were supported by infection assays in C3H/HeJ mice, which have a natural point mutation in the *TLR4* gene, finding that they were deficient in the production of antimicrobial mediators, such as H_2_O_2_, when challenged with lipid extracts from *S. schenckii* yeast-like cells (Carlos et al., 2009). The *TLR4* role during *S. brasiliensis* sensing has also been assessed. The tlr4 ^−^/^−^ knockout mice infected with this fungal species showed high levels of fungal burden and deficient cytokine production after 14 days of infection when compared with wild-type mice, indicating that this receptor is also relevant for this pathogen control (Rossato et al., 2019). The TLR2 contribution to the innate immune response against *S. schenckii* has also been investigated. The tlr2 -/- knockout mice showed a lower percentage of macrophages with phagocytized yeasts-like cells when compared with cells from wild-type animals (Negrini Tde et al., 2013).

In addition to TLR2 and TLR4, dectin-1 plays an important role in the *Sporothrix* recognition. The fungal β-1,3-glucan recognition by dectin-1 induces a proinflammatory response and neutrophils and macrophages activation (Taylor et al., 2007). After inoculation of *S. schenckii* yeast-like cells in the peritoneal space and a follow up of 2 weeks, peritoneal macrophages showed that dectin-1 expression was significantly upregulated, as well as cytokine production, suggesting an important role for this receptor during the establishment of an immune response during sporotrichosis (Jellmayer et al., 2017).

Like other pathogens, the *S. schenckii* cell wall contains polysaccharides, and their abundance and distribution within the wall have important roles in modulating the animal immune response (Lozoya-Pérez et al., 2020; Villalobos-Duno et al., 2021). The effect of *S. schenckii* chitin-rich heteroglycan on the immune response was assessed by histopathological analyses of skin lesions in a mouse model of sporotrichosis, and the phagocytic function and cytokine production by peritoneal macrophages were evaluated (Huang et al., 2021). Following the inoculation of chitin in BALB/c mice, the granulomatous inflammation was reduced within 5 weeks, and fungal phagocytosis and TNF-α production were significantly improved (Huang et al., 2021).

A 70-kDa cell wall glycoprotein has been reported as an antigen that induces high antibody titers, being specially recognized by IgG1 and IgG3 in murine models of systemic sporotrichosis (Nascimento and Almeida, 2005). Moreover, this protein facilitates yeast-like cell adhesion to the mouse tail dermis, suggesting a role in *S. schenckii* pathogenesis (Ruiz-Baca et al., 2009). Passive immunization of mice with a monoclonal anti-Gp70 antibody before inoculation of a lethal dose of *S. schenckii* showed a significant reduction of colony-forming units in the spleen and liver of immunized mice, possibly due to an antibody-driven inhibition of *S. schenckii* adhesion to host tissues (Nascimento et al., 2008). The monoclonal antibody was protective regardless of the *Sporothrix* species, as demonstrated by its effectivity in intraperitoneally infected mice with *S. brasiliensis* (de Almeida et al., 2015). However, antibody humanization was necessary to avoid the production of anti-murine antibodies in patients (de Almeida et al., 2017). This monoclonal antibody showed efficiency in the control of sporotrichosis *in vitro* and *in vivo*, representing a great alternative for treatment in humans (de Almeida et al., 2017).

In addition, the model of systemic infection in mice can contribute to the study of antifungal drugs against *Sporothrix*. The conventional approach to treat sporotrichosis includes administration of potassium iodide, amphotericin B, azoles (itraconazole, fluconazole), or terbinafine (Orofino-Costa et al., 2017). Previous studies suggested that amphotericin B can prevent death in mice with disseminated infection and reduce the number of clinical lesions (Tsubura and Schwarz, 1961; Kan and Bennett, 1988). This antifungal drug evoked the best reduction of animal mortality without any adverse effects, and better performance when compared with azoles, which significantly reduced animal mortality, without healing the tail and paw lesions were persistent in almost every mouse (Kan and Bennett, 1988). Posaconazole has also been studied *in vivo* to treat infections caused by either *S. schenckii* or S. *brasiliensis*, using a murine model of disseminated sporotrichosis, demonstrating a good efficacy in fungal burden reduction without toxic effects (Fernández-Silva et al., 2012a). A later study reported the *in vitro* synergistic combination of posaconazole with amphotericin B to treat murine sporotrichosis, showing that administration of both drugs to mice with systemic infection led to 100% survival rates (Mario et al., 2015).

Both the systemic and peritoneal infections in murine models have contributed to the understanding of the mechanisms involved in the host immune response during sporotrichosis, establishing the foundations for the understanding of the *Sporothrix*-immunity interaction in other mammalians, such as human beings.

### Subcutaneous Model of Sporotrichosis in Mice

Since sporotrichosis is the most frequent subcutaneous mycosis in Latin America (Chakrabarti et al., 2015), it is essential to understand the mechanisms behind *Sporothrix* spp. pathogenesis in the subcutaneous tissue. The subcutaneous inoculation reflects the natural infection route for these fungal species and takes into account the traumatic inoculation with soil and plant debris contaminated or the animal-driven implantation. The subcutaneous infection in mice has been carried out in the hind footpad or the dorsal sacral region, using either conidia or yeast-like cells (Tachibana et al., 1999; Castro et al., 2013). The subcutaneous infection is followed up by weight measurement, footpad thickness, the evolution of the primary skin lesion, and the development of secondary lesions (de Almeida et al., 2018). The progression from subcutaneous to a systemic infection was demonstrated in BALB/c mice subcutaneously inoculated into the left hind footpad with *S. schenckii* yeast-like cells. After this challenge, animals showed weight loss, cutaneous lesions, inactivity, and different survival rates, mimicking the human disease (Brito et al., 2007). Histological analyses revealed inflammatory infiltrates and granuloma in the liver and an inflammatory reaction in the inoculation area (Brito et al., 2007).

It is well-established that *S. brasiliensis* is related to the zoonotic transmission of sporotrichosis and the emergence of severe cases of this disease (Rodrigues et al., 2016). To investigate whether *S. schenckii* and *S. brasiliensis* share similar virulence factors and whether they have differences in pathogenicity, clinical strains of both species were subcutaneously inoculated in mice and the disease progression was followed by the evolution of the primary skin lesion, secondary lesions development, and dissemination to spleen and lungs (Castro et al., 2013). The study showed that *S. brasiliensis* clinical isolates had increased pathogenicity when compared with *S. schenckii*, with persistent skin lesions in mice and a higher ability to disseminate, with significant fungal load observed in the lungs and spleen, that lead to 100% mortality of infected mice (Castro et al., 2013). In agreement with these results, another study showed that subcutaneously challenged mice with *S. brasiliensis* developed lesions in the footpad 1 week post-infection, showing an ulcerous-crust appearance similar to that seen in both human and animal sporotrichosis (Della Terra et al., 2017). In addition, it was shown that this pathogen can disseminate across the central nervous system, even when a subcutaneous route is used (Della Terra et al., 2017).

In this context, differences between immune responses against *S. schenckii* and *S. brasiliensis* have been reported. The role of cellular immune responses in sporotrichosis was investigated by evaluating T helper, and T regulatory cells (Tregs), a subset of T lymphocytes with a FoxP3+ phenotype. Either *S. schenckii* or *S. brasiliensis* were subcutaneously inoculated in the dorsal sacral region of mice, and infection progression was monitored by observing fungal load in skin lesions, spleen, and liver, thus evaluating both local and systemic infection. It was demonstrated that *S. brasiliensis* developed more extensive local lesions and was also more efficient in colonizing organs than *S. schenckii*, confirming a higher virulence for *S. brasiliensis* (Batista-Duharte et al., 2018b). This difference was related to the cellular immune response: while *S. schenckii* infection was controlled by T helper cells (Th1 and Th17) and associated with late Tregs response, the *S. brasiliensis* infection evoked poor Th1 response at early stages of infection, with higher Th17 and Tregs in the advanced phase (Batista-Duharte et al., 2018b). These results led to hypothesize that Tregs probably promotes a deleterious effect in the protective immune response mediated by Th1 lymphocytes while promoting a compensatory Th17 response. To elucidate the role of Tregs cells, DEREG (DEpletion of REGulatory T cells) mice were used to establish a subcutaneous infection caused by *S. schenckii*. These mice express proteins that are under the control of the FoxP3 locus, allowing the depletion of FoxP3+ Tregs at any stage of the disease (Batista-Duharte et al., 2020). Tregs depletion led to an enhanced Th1 response, whereas no significant differences were seen in the Th17 population between depleted and no depleted-Tregs animal groups (Batista-Duharte et al., 2020). In the late infection stage, Tregs depletion correlated with a beneficial effect in fungal clearance, suggesting that Th1 and Th17 stimulation after Tregs deletion helped to a faster fungal elimination (Batista-Duharte et al., 2020). As in the systemic and intraperitoneal infection, cellular and humoral immune responses triggered upon *Sporothrix* infection into the subcutaneous tissue may play important roles in the sporotrichosis development and severity (Verdan et al., 2012). Animals infected with *S. brasiliensis* induced antibody production against a wide diversity of antigens during the subcutaneous infection in mice, and these antibodies showed high cross-reactivity levels when tested against *S. schenckii* antigenic preparations, suggesting conservation of epitopes in both species (Della Terra et al., 2017).

Because of all the above-described examples, it is clear that the subcutaneous model of sporotrichosis in mice can serve as a reference to study *Sporothrix* pathogenesis and host immune responses, contributing to the understanding of the mechanisms involved in the subcutaneous infection.

### Other Mammals

In addition to mice, other mammals such as rats, rabbits, hamsters, guinea pigs, and cats have been used as a host for experimental infections with *S. schenckii* (Charoenvit and Taylor, 1979). Rats can be intraperitoneally infected with *S. schenckii*, generating a systemic infection that allows cytokines quantification and histological analyses of organs such as the liver, spleen, and testicles (Castro et al., 2016, 2017). The immune anti-*Sporothrix* response developed by the host can also be investigated in rats. Mast cells are a source of histamine and proteases and are important effector cells in the protective immunity against pathogens (Saluja et al., 2012). Romo Lozano et al. aimed to evaluate the *in vitro* mast cells response to *S. schenckii* yeasts-like cells, using mast cells from Wistar rats peritoneal exudates. These fungal cells stimulated morphological changes in mast cells, which are related to activation, and produced increased levels of TNF-α and IL-6 (Romo-Lozano et al., 2012, 2014). It is established that oxidative stress plays an important role in many fungal infections (Pohanka, 2013). In a systemic model of sporotrichosis in rats, it was found that lipid peroxidation, catalase, and superoxide dismutase activities were significantly increased in infected animals when compared to control healthy rats (Castro et al., 2017). These results strongly suggest that the intense inflammatory response in sporotrichosis could be due to a redox imbalance, causing high amounts of reactive oxygen species that increase tissue damage, as seen in infected rats, which developed granulomatous lesions in the inoculation sites (Castro et al., 2017).

Rats are a useful model to assess the antifungal activity of compounds against sporotrichosis. Terbinafine and itraconazole have demonstrated effectiveness in reducing clinical lesions in *Sporothrix*-infected rats, such as nodular and whitish lesions on the spleen, liver, and testicles, suggesting efficacy to treat systemic sporotrichosis (Meinerz et al., 2008).

Guinea pigs had been used in the past to study antifungal compounds against sporotrichosis (Van Cutsem et al., 1987). However, there are no reports of using this model to assess *S. schenckii* pathogenesis. Nevertheless, this model is frequently used in the investigation of *Candida albicans* and *Cryptococcus neoformans* virulence (Murphy et al., 1974; Sohnle and Kirkpatrick, 1977; Odds et al., 2000). The lack of use of this model to study sporotrichosis might be due to the availability of other *in vivo* models that have more advantages, such as easier manipulation and the examination of larger animal groups.

Experimental sporotrichosis in cats can resemble in many ways human infection. Once animals are inoculated, they develop primary local lesions, followed by secondary lesions in the lymphatic nodes, like in human sporotrichosis, in which lesions frequently extend to lymphatic vessels (Barbee et al., 1977). Cats are important models in the study of antifungal drugs for sporotrichosis treatment. Itraconazole and ketoconazole had been tested *in vivo* in sick cats to determine the effectiveness and safety, and have shown good responses to the antifungal treatment (Schubach et al., 2004). However, the occurrence of adverse effects might happen during treatment, as seen in the *in vivo* assays, where anorexia, vomiting, and diarrhea were frequent in the tested cats (Schubach et al., 2004; Pereira et al., 2010).

Even though it is not regarded as an experimental model of sporotrichosis, the study of naturally infected cats is useful in epidemiological studies. For example, the study of 300 cats, including healthy and *Sporothrix*-infected animals showed that healthy cats have a minor role in sporotrichosis transmission (Macêdo-Sales et al., 2018).

## Invertebrate Models to Study Sporotrichosis

As mentioned before, animal models are a key tool for virulence evaluation of human pathogenic fungi, being the murine model the gold standard. However, some concerns about ethical issues and animal welfare have motivated the limitation of animal experimentation and the search for alternative models, such as invertebrates, where ethical concerns are less restrictive and because of their life cycle, several individuals can be included in the experimental groups, strengthening the statistical power (Jacobsen, 2014; Singulani et al., 2018). Invertebrate models, such as *Galleria mellonella, Tenebrio molitor*, and *Acanthamoeba castellani* have been successfully used to study the *Sporothrix*-host interaction (see Table 1).

### Galleria mellonella

Larva of the wax moth, *G. mellonella*, has been extensively used in the study of human fungal pathogens, such as *Aspergillus fumigatus, Candida* spp., *Cryptococcus* spp., *Paracoccidioides* spp., *Histoplasma capsulatum*, and *Sporothrix* spp. (Brennan et al., 2002; Mylonakis, 2008; Clavijo-Giraldo et al., 2016; Navarro-Arias et al., 2016; Perez-Garcia et al., 2016; Hernandez-Chavez et al., 2018, 2019; Singulani et al., 2018; Garcia-Carnero et al., 2020). This model has several advantages when compared to mammals, such as easier manipulation, the requirement of simple facilities for housing and breeding, cheaper to purchase and to maintain, they can be kept at 37°C and the fact that they can generate results in just a couple of weeks (Mylonakis, 2008; Pereira et al., 2018; Garcia-Carnero et al., 2020). In addition, the *G. mellonella* immune system is structurally and functionally similar to mammalian innate immunity (Kavanagh and Reeves, 2004; Malavia et al., 2020). Virulence in this model can be measured easily by the melanization and subsequent insect death (Mylonakis, 2008; Pereira et al., 2018). Also, changes in the hemocyte density, phenoloxidase activity, melanization, and measurement of free lactate dehydrogenase are used as indicative of virulence and the immune response against the fungal pathogen (Bergin et al., 2006; Kavanagh and Sheehan, 2018; Garcia-Carnero et al., 2020).

The *G. mellonella* model is suitable for the study of the *Sporothrix* species, as highlighted in different reports. First, it has been demonstrated that larva infection with conidia, germlings, and yeast-like cells from *S. schenckii* and *S. brasiliensis* killed the larvae in a dose-dependent manner, being the yeast-like morphology the one capable to reproduce the result generated in mice (Clavijo-Giraldo et al., 2016). In addition, it was observed that the concentration 1 x 10^5^ cells/μL caused a difference in the mortality rate, while no difference was observed when higher fungal loads were inoculated, which could be explained by an exaggerated immune response at high fungal concentrations, being a detrimental factor for the animals' fitness (Clavijo-Giraldo et al., 2016). Similar to the mammalian host, the ideal temperature to maintain the yeast-like morphology in these experiments is 37°C (Clavijo-Giraldo et al., 2016). Finally, it has been observed that infection with *S. brasiliensis* yeast-like cells was more aggressive when compared with animals inoculated with *S. schenckii*, similar to what has been reported in the murine model (Clavijo-Giraldo et al., 2016; Della Terra et al., 2017). The virulence of different strains from *S. schenckii* and *S. brasiliensis* was also evaluated in this model. The already reported *S. schenckii* low virulent strains 1099-18 ATCC MYA 4821, Ss39, and Ss47, and the virulent strain SS-B02 were used to infect larvae and it was observed that the SS-B02 and Ss39 strains were the most virulent ones in *G. mellonella* (Clavijo-Giraldo et al., 2016). The strain Ss39 had been reported to be a low virulent strain in mice, and the fact that this strain showed a higher virulence in larvae could be explained by the inoculum preparation in different carbon sources (Clavijo-Giraldo et al., 2016; Lozoya-Pérez et al., 2020). In addition, similar experiments were performed with *S. brasiliensis*, and it was observed that the strains 5110 ATCC MYA 4823 and HUPE 114158 were the most virulent in *G. mellonella*, while HUPE 114500 and UFTM01 had mild virulence, these results were similar to those generated in the subcutaneous model of sporotrichosis in mice (Clavijo-Giraldo et al., 2016).

This model was also used to evaluate the virulence of *S. schenckii* mutants. Mutants with different degrees of *OCH1* silencing, a Golgi α1,6-mannosyltransferase with an important role in the *N*-linked glycans synthesis, had a virulence attenuation in both *G. mellonella* and mice (Lozoya-Perez et al., 2019). In addition, silenced mutant strains in *RlmD*, a gene that encodes an epimerase/reductase enzyme involved in the UPD-rhamnose synthesis, showed virulence attenuation in *G. mellonella* and induced low cytotoxicity, hemocyte, and phenoloxidase levels (Tamez-Castrellón et al., 2021). Since similar fungal burdens were found in the hemolymph of both wild-type and mutant strains, the reduction in the mortality rate was associated with changes in the virulence rather than the ability of mutant cells to adapt to the host milieu (Tamez-Castrellón et al., 2021). Mutant cells that express the green fluorescent protein but with no gene silencing involved have also been assessed in this alternative model of sporotrichosis (Lozoya-Perez et al., 2018).

The effect of the culture medium to generate fungal biomass on virulence was also tested using *G. mellonella* larvae. The kill curves for *S.schenckii, S. brasiliensis*, and *S. globosa* were similar to those reported in mice, being S. *brasiliensis* the most virulent species, followed by *S. schenckii* and *S. globosa*, when cells were grown in YPD or brain-heart infusion (Lozoya-Pérez et al., 2020). It was also found that carbon or nitrogen limitation impaired *S. schenckii* and *S. brasiliensis* abilities to kill the larvae, but not for *S. globosa* (Lozoya-Pérez et al., 2020). This observation was linked to an increase in hemocyte countings, phenoloxidase activity, and lower cytotoxicity, as a consequence of alterations in cell wall composition and organization in *S. schenckii* and *S. brasiliensis*, with a significant increment in the β-1,3-glucan at the cell surface (Lozoya-Pérez et al., 2020). This was not observed for *S. globosa*, since this species has naturally higher levels of β-1,3-glucans exposed at the cell surface, regardless of the culture medium to prepare yeast-like cells (Lozoya-Pérez et al., 2020). The exposure of this cell wall polysaccharide increased the ability of the insect hemocytes to uptake and degrade fungal cells, explaining the changes in virulence of cells growing in limited carbon and nitrogen conditions (Lozoya-Pérez et al., 2020).

In *G. mellonella*, like in other insects, the immunological priming is a response similar to the immunological memory found in mammals (Contreras-Garduño et al., 2015). *S. schenckii* Gp70 was heterologously expressed in bacteria and the recombinant protein ability to induce immunological priming was assessed (Martinez-Alvarez et al., 2019). When *G. mellonella* larvae were inoculated with increasing concentrations of recombinant Gp70 before a lethal challenge with *S. schenckii*, the mortality rate decreased. Also, an increment in the hemocyte population and phenoloxidase activity was observed with concentrations of 40 μg recombinant Gp70 and higher (Martinez-Alvarez et al., 2019). Similar results were recently reported for Hsp60 and Pap1, proteins that are part of cell wall peptidorhamnomannan (García-Carnero et al., 2021). Besides the ability of the recombinant versions of these proteins to induce immunological priming, preincubation of yeast-like cells with anti-Hsp60 or anti-Pap1 antibodies resulted in cells incapable of killing *G. mellonella*, indicating a relevant role of both proteins in the *S. schenckii*-*G. mellonella* interaction (García-Carnero et al., 2021).

This model has also been used to assess the effect of antifungal drugs on *Sporothrix* spp. Itraconazole-loaded nanostructured lipid carriers showed no toxicity for larvae and improved the survival rate of insects infected with *S. brasiliensis* yeast-like cells, with 100% of animals surviving at the end of the 5-day observation period (Passos et al., 2020). The effect of buparvaquone, an antiprotozoal hydroxynaphthoquinone drug, on *S. brasiliensis*-induced infection was also assessed. Larvae inoculated with a single dose of 5 mg/kg buparvaquone had an increased survival rate than the control group, and the drug performance was even better than that observed with itraconazole (Borba-Santos et al., 2021).

### Tenebrio molitor

The mealworm beetle, *T. molitor*, is another popular invertebrate model that has been used for the study of different pathogenic fungi, such as *C. albicans, C. neoformans, Fonsecaea pedrosoi*, and *Fonsecaea monophora* (de Souza et al., 2015; Canteri de Souza et al., 2018; Fornari et al., 2018). This model shares all the advantages already mentioned for *G. mellonella*, but in addition, it generates bigger larvae which make it easier to manipulate and obtain higher hemolymph volumes to analyze different physiological parameters (Canteri de Souza et al., 2018; Vigneron et al., 2019). When this model was assessed for the analysis of *S. schenckii, S. brasiliensis*, and *S. globosa* infection, the same virulence pattern as for the mice model and *G. mellonella* was observed, being *S. brasiliensis* the most virulent species and *S. globosa* the least (Lozoya-Perez et al., 2021). These findings were validated using mutants with different virulence degrees, observing that low virulent strains stimulated low cytotoxicity and immune response (phenoloxidase activity and hemocyte counting), and therefore a longer larvae survival; while the highly virulent strains caused an exacerbated immune response, with high hemocytes levels. In addition, immunological priming that protected the larvae was observed when the recombinant Gp70 was inoculated before a lethal challenge with *S. schenckii* (Lozoya-Perez et al., 2021). All of these results suggest that *T. molitor* is a good model as *G. mellonella* for the evaluation of *Sporothrix* spp. virulence.

### Acanthamoeba castellanii

*A. castellanii* is an environmental soil amoeba that feeds on bacteria and fungi by phagocytosis (Chambers and Thompson, 1976). This non-conventional model was also found to be useful for the study of *Blastomyces dermatitidis, H. capsulatum*, and *Sporothrix* virulence, due to its phagocytic features, which can be compared to those of mammalian macrophages (Steenbergen et al., 2004; Singulani et al., 2018). It was reported that the interaction between *S. schenckii* and *A. castellanii* caused amoeba death due to the fungal growth, which uses *A. castellanii* as a nutritional source under starvation conditions (Steenbergen et al., 2004). It was also observed that *S. schenckii* grew as hypha and pseudohyphae inside the amoeba, very similar to what has been observed in macrophages, and electron microscopy revealed that the fungus gets enclosed in a membrane-bound vacuole after phagocytosis (Steenbergen et al., 2004). In addition, when phagocytosis assays were performed with macrophages and *A. castellanii*, the rate of yeast-like cells internalization was similar for both models (Steenbergen et al., 2004).

## *Ex vivo* Models for the *SPOROTHRIX* spp.-Host Interaction

The establishment of protocols aiming to reduce animal use in experimentation has become a worldwide trend, and currently, there has been an emphasis on the application of the 3Rs, that is, replacement, reduction, and refinement, which can be beneficial for good science, as well as for animal welfare (MacArthur Clark, 2018; Brilhante et al., 2021). The *ex vivo* models refer to the use of living tissues or cells, which are recovered from an organism, to apply them in experiments to be carried out in artificial environments (Ohnemus et al., 2008; Maciel Quatrin et al., 2019). These models maintain the cell types and the format of the infected tissues without the need of using animals (Lossi and Merighi, 2018). *Ex vivo* experimental models, such as endothelial cells, immune cells, and cell lines, can simulate infectious processes occurring *in vivo* and are of great importance to evaluate new drugs, learn more about pathogenesis, virulence, immune response, and the fungal ability to adhere and form biofilms (Ohnemus et al., 2008; Maciel Quatrin et al., 2019; Brilhante et al., 2021; see Table 1).

### Endothelial Cells

Many fungi that cause invasive diseases invade host epithelial cells, affecting the mucosa and subsequently invading endothelial cells during the dissemination stage (Filler and Sheppard, 2006). *C. albicans* and *C. neoformans* adhere to and penetrate the endothelial compartment, suggesting that the endothelium is an important barrier that must be overcome by pathogens (Klotz, 1992). Little is known about this interaction during sporotrichosis, but some studies support that *Sporothrix* spp. also crosses this barrier (Lima et al., 1999; Figueiredo et al., 2004). Thus, implementing the *ex vivo* use of endothelial cells could help to understand this phenomenon. Human umbilical vein endothelial cells have been used to determine the relevance of these cells during *S. schenckii* infections (Figueiredo et al., 2004). The analyses indicated that the yeast-like cells are in the interendothelial junctions, internalized within endocytic vacuoles after 2 h of interaction, and 24 h post-interaction no damage was observed in the endothelial cells (Figueiredo et al., 2004), meaning that *S. schenckii* is able to adhere and invade these cells without affecting cell viability.

### Macrophages

Currently, using different primary immune cells it is possible to assess the contribution of immune effectors in the *Sporothrix*-host interaction. Macrophages, as innate immune cells, depend on pattern recognition receptors to engage with pathogens and are one of the most important defense lines against fungal pathogens, including *Sporothrix* spp. (Netea et al., 2008; Carlos et al., 2009; Martinez-Alvarez et al., 2014). Pattern recognition receptors in macrophages such as TLRs, the family of nucleotide-binding oligomerization domain-like receptors (NOD and NLR), the NLRP3 domain, and C-type lectins, like dectin-1, have been studied during the macrophage-*Sporothix* spp. interplay. *Ex vivo* tests with peritoneal macrophages have shown a correlation between the TLR4 expression and the secretion of pro- and anti-inflammatory mediators, such TNFα and IL-10, respectively, during sporotrichosis (Sassá et al., 2009). Interestingly, even though the *Sporothrix* cell wall contains significant mannose levels (Martinez-Alvarez et al., 2017; Lopes-Bezerra et al., 2018b; Villalobos-Duno et al., 2021), it has been shown that mannose receptor has a minor contribution in the cytokine production in *ex vivo* models (Martinez-Alvarez et al., 2017). In an *ex vivo* sporotrichosis model using peritoneal exudate cells challenged with *S. schenckii* cell wall peptide-polysaccharide complex, M2 macrophages were the most predominant population, and expressed arginase-I activity peaks as well as IL-10 and transforming growth factor-beta (Alegranci et al., 2013).

Macrophages derived from human peripheral blood mononuclear cells (PBMCs), allow determining the interaction of pathogens with the host, and the contribution of the phagocytic processes in the defense mechanisms. This model has been used to analyze the relevance of *S. schenckii* protein glycosylation to pathogenesis. Loss of the *N*-linked glycan outer chain, by *OCH1* silencing, led to significant changes in the cell wall composition and structure, which negatively affected phagocytosis by PBMC-derived macrophages (Lozoya-Perez et al., 2019). Similar results have also been found in *C. albicans* and other *Candida* species (Cambi et al., 2008; McKenzie et al., 2010; Navarro-Arias et al., 2016; Perez-Garcia et al., 2016; Gonzalez-Hernandez et al., 2017; Hernandez-Chavez et al., 2019). Moreover, the *S. schenckii*-PBMC-derived macrophage interaction was significantly affected by the *RlmD* silencing, a key gene in the UDP-rhamnose synthesis (Tamez-Castrellón et al., 2021). These analyses led to the observation that rhamnose immune sensing is a TLR4-dependent process (Tamez-Castrellón et al., 2021).

### Human Peripheral Blood Mononuclear Cells

Many works have shown that antifungal immunity requires the orchestration of both innate and adaptive immune responses. PBMCs represent an attractive source of innate and adaptive immune cells because they can be easily obtained from patients and healthy donors. One disadvantage of working with these cells is that they are sensitive to variables such as temperature, stimulation, and collection time (Acosta Davila and Hernandez De Los Rios, 2019). This model has been used to assess the relevance of the *S. schenckii* and *S. brasiliensis* cell wall composition during the interaction with innate immune cells (Martinez-Alvarez et al., 2017). By measuring the stimulation of both pro- and anti-inflammatory cytokines by PBMCs, it was established that *S. schenckii* stimulates higher levels of pro-inflammatory cytokines, compared to *S. brasiliensis*, which stimulated higher IL-10 levels (Martinez-Alvarez et al., 2017). The dectin-1 receptor was key for cytokine production and TLR2 and TLR4 participated in the detection of both *Sporothrix* species (Martinez-Alvarez et al., 2017). From these results, it is possible to suggest that in the case of *S. brasiliensis*, a pro-inflammatory scenario was less stimulated by conidia, and as a consequence, this fungus has a better ability to colonize tissues and establish the infective process (Martinez-Alvarez et al., 2017). This could help yeast-like cells to spread easily within the lymphatic system and deep organs (Martinez-Alvarez et al., 2017). In addition, it has been found that there are other soluble receptors involved in the recognition of *Sporothrix* pathogenic species, such as PTX3. Secretion of this receptor facilitates the deposition of some complement system components, helping in the pathogen elimination. It was also demonstrated that CR3, a complement receptor expressed in macrophages, directly recognizes peptidorhamnomannan from both *S. schenckii* and *S. brasiliensis* (Neves et al., 2021). This model has also been used to assess the contribution of protein glycosylation to the *S. schenckii*-immune effectors interaction. Loss of proper *N*-linked glycosylation or rhamnosylation led to changes in the ability to stimulate TNFα, IL-1β, IL-6, and IL-10 by PBMCs (Lozoya-Perez et al., 2019; Tamez-Castrellón et al., 2021).

### Mast Cells

Mast cells are a useful tool to elucidate the role of cutaneous defense mechanisms in sporotrichosis. When bone-marrow-derived mast cells were incubated with *S. schenckii* yeast-like cells no cell degranulation was observed, but there was an increase in IL-6 and TNFα stimulation, an observation that fits well with the *in vivo* model of sporotrichosis in rats, where the fungus promotes skin inflammation and overproduction of both cytokines, thus exacerbating cutaneous sporotrichosis (Romo-Lozano et al., 2014; Jiao et al., 2020). Mast cells can detect *S. schenckii* through TLR2, TLR4, and C-type receptors (Sandig and Bulfone-Paus, 2012).

### Dendritic Cells

Dendritic cells (DCs) are known as specialized antigen-presenting cells and are an important innate immunity component that recognizes pathogen patterns and can activate an adaptive response, inducing antifungal immunity (Verdan et al., 2012; Ikeda et al., 2018). These cells can be used as *ex vivo* models to study *Sporothrix* spp. *S. brasiliensis*, like other fungal species, can release extracellular vesicles that interact with host cells and modulate the host's immune response (Ikeda et al., 2018). In particular, these extracellular vesicles induce an increase in the DCs phagocytosis index, and the production of IL-12 and TNFα (Ikeda et al., 2018), cytokines that perform very important immunoregulatory functions in the host defense. These characteristics can favor the fungus to mediate its establishment in the host. In addition, DCs are capable of recognizing information from fungal pathogens and translating it into differential T-cell responses. The mechanisms involved in the interaction of *S. schenckii* and DCs are not completely clear. *S. schenckii* yeast-like cells activated DCs and made them capable of triggering T cell responses, promoting IFN-γ production (Verdan et al., 2012). A yeast-derived exoantigen from this fungal species was capable of also activating DCs, to promote IFN-γ production, and in addition, the production of IL-17, with the consequent activation of a Th17 inflammatory response, demonstrating the plasticity of DCs in translating data associated with *S. schenckii* and its exoantigen into T-cell differential responses (Verdan et al., 2012).

### Cell Lines

Cell lines have the advantage of being easy to use and providing highly reproducible results (Last et al., 2021). These can be cultured for a limited time or can be immortalized, and allow extensive control over growth conditions, O_2_ and CO_2_ saturation, temperature, pH, and nutrients (Last et al., 2021). There are many cell lines available, and represent a valuable resource that allows the study of conserved mechanisms in antifungal immune responses (Last et al., 2021), and the study of possible molecules with antifungal properties (Borba-Santos et al., 2020). For the latter, a Pathogen Box library for compounds with potential activity against *S. brasiliensis* and *S. schenckii* was analyzed and used to assess cytotoxicity potential (Borba-Santos et al., 2020). The most promising compounds, MMV102872, and iodoquinol were tested at various concentrations in the mammalian epithelial cell line LLC-MK2 (ATCC CCL-7), and the concentrations that caused 50% cytotoxicity of MMV102872 and iodoquinol were 8.7 M and 5 M, respectively, resulting in a high index of the selectivity of these compounds for *S. schenckii* and *S. brasiliensis* (Borba-Santos et al., 2020). Other cell lines have also been used for toxicity studies of these compounds, such as the HepG2 cell line, where reduced toxicity was also observed. These cell lines may be key to the study of compounds that may be candidates for the sporotrichosis treatment (Borba-Santos et al., 2020).

The human monocytic cell line THP-1 has also been used to determine phagocytosis of *S. schenckii* conidia, which were opsonized with human serum components. It was observed that the uptake of opsonized conidia stimulated ROS production, which led to fungal death (Guzman-Beltran et al., 2012). TNFα release was not stimulated by opsonized or non-opsonized conidia, but a differential cytokine production was observed when yeast-like cells were opsonized (Guzman-Beltran et al., 2012). These results obtained with the cell line have recently been confirmed using human monocyte-derived macrophages (Guzman Beltrán et al., 2021).

Epithelial cells are a model that can be used to evaluate the *S. schenckii* proteolytic activity (Sabanero López et al., 2018). By using the L929 epithelial cell line, it has been shown that *S. schenckii* has an extracellular proteolytic activity that allows the pathogen to penetrate and colonize the epithelium, through a paracellular route, and act on the cytoskeleton fibers during the infection process; suggesting a possible action of proteases on actin, altering the morphology and integrity of the epithelial cells (Schindler and Segal, 2008; Sabanero López et al., 2018). These effects on epithelial cells have also been observed using alveolar cells infected with *A. fumigatus* (Kogan et al., 2004).

### Feline Claw Fragments

Cat scratches are a major event by which sporotrichosis can be transmitted. *Sporothrix* spp. has been isolated from cats, but there is a lack of knowledge about how this fungus colonizes felines and its ability to form biofilms (Gremião et al., 2017; Brilhante et al., 2021). For this reason, using *ex vivo* models such as cat's claw fragments, it has been possible to elucidate this information. Strains of *S. brasiliensis, S. schenckii, S. globosa*, and *Sporothrix mexicana* isolated from feline, human and environmental sources, were used to analyze fungal ability to form biofilms on cat's claw fragments, obtained from domestic cats of both sexes, older than 1 year, which did not show clinical signs of skin lesions (Brilhante et al., 2021). For *S. brasiliensis* and *S. globosa*, more characteristic and better-elaborated biofilms were formed, while in *S. schenckii* the biofilm formation consisted of weakly associated hyphae, which could indicate a lower amount of matrix material compared to the other species (Brilhante et al., 2021).

## Concluding Remarks

The availability of different mouse strains, infection routes, and administration of pharmacotherapies represent great advantages for the murine models of sporotrichosis over other alternatives currently available. However, the strict ethical concerns and regulations, along with the requirement of large animal populations to be enrolled in sophisticated experimental approaches, have focused the attention of the specialized community on invertebrates and *ex vivo* models. Besides the vertebrate models already used to study *Sporothrix* spp. pathogenesis, zebrafish is a well-accepted model in medical mycology (Rosowski et al., 2018). Because of its particularities, live imaging to study the pathogen dissemination within the host and interaction with immune cells are advantages that offer these organisms over other vertebrates (Gomes and Mostowy, 2020). The invertebrates repertoire to experimentally study sporotrichosis can be expanded and include other popular alternative hosts to study fungal pathogenesis, such as *Drosophila melanogaster* and *Caenorhabditis elegans* (Chamilos et al., 2007). They have the advantage of having their genomes are already available, and for the case of flies, mutant strains in key immune regulators are available (Limmer et al., 2011). However, since these organisms are not viable at 37°C (Limmer et al., 2011; Singulani et al., 2018), it is unlikely that they might be useful to dissect *Sporothrix* pathogenesis and virulence. For the case of *ex vivo* experimental settings, 3D models represent an attractive and sophisticated alternative to study sporotrichosis in a system that resembles the natural architecture of the host infected tissues. Skin 3D models have been used to study several infectious diseases, including oral candidiasis (Tabatabaei et al., 2020). The basic skin model consists of dermal fibroblast and keratinocytes, followed by immune cells below the tissue, which can finally be infected with the fungal pathogen, which mimics fungal infections and allows the study of host-pathogen interaction (Kühbacher et al., 2017). This contrasts with the primary or cell line cultures that form monolayers. Even though this is a promising model to study sporotrichosis, the cost associated with this experimental approach and the inability to establish a systemic model of infection (Tabatabaei et al., 2020), are disadvantages that should be taken into consideration before implementation. The multi-organ-on-chip is another *ex vivo* alternative that remains to be used to study sporotrichosis. This alternative has been used to analyze the infection caused by *C. albicans* and provides the opportunity to know how fungal dissemination is carried out within organs (Last et al., 2021).

In summary, several alternatives to study the host-*Sporothrix* interaction are currently available, and there are factors to take into account before choosing the most appropriate one. Finally, the combination of models is likely to provide a robust dataset and to exploit the unique advantages of each of the models.

## Author Contributions

AV-M, MG-G, LG-C, and HM-M conceived the literature revision, retrieved information from databases, analyzed information, drafted the manuscript, and approved the final version of it. All authors contributed to the article and approved the submitted version.

## Funding

This work was supported by Consejo Nacional de Ciencia y Tecnología (ref. FC 2015-02-834 and Ciencia de Frontera 2019-6380) and Red Temática Glicociencia en Salud (CONACYT-México).

## Conflict of Interest

The authors declare that the research was conducted in the absence of any commercial or financial relationships that could be construed as a potential conflict of interest.

## Publisher's Note

All claims expressed in this article are solely those of the authors and do not necessarily represent those of their affiliated organizations, or those of the publisher, the editors and the reviewers. Any product that may be evaluated in this article, or claim that may be made by its manufacturer, is not guaranteed or endorsed by the publisher.
